# What Patients Value in Physicians: Analyzing Drivers of Patient Satisfaction Using Physician-Rating Website Data

**DOI:** 10.2196/13830

**Published:** 2020-02-03

**Authors:** Sonja Bidmon, Ossama Elshiewy, Ralf Terlutter, Yasemin Boztug

**Affiliations:** 1 Department of Marketing and International Management Alpen-Adria-Universitaet Klagenfurt Klagenfurt am Woerthersee Austria; 2 Department of Business Administration, Marketing and Consumer Behavior University of Goettingen Goettingen Germany

**Keywords:** online physician ratings, patient satisfaction, multiattribute models, health care management

## Abstract

**Background:**

Customer-oriented health care management and patient satisfaction have become important for physicians to attract patients in an increasingly competitive environment. Satisfaction influences patients’ choice of physician and leads to higher patient retention and higher willingness to engage in positive word of mouth. In addition, higher satisfaction has positive effects on patients’ willingness to follow the advice given by the physician. In recent years, physician-rating websites (PRWs) have emerged in the health care sector and are increasingly used by patients. Patients’ usage includes either posting an evaluation to provide feedback to others about their own experience with a physician or reading evaluations of other patients before choosing a physician. The emergence of PRWs offers new avenues to analyze patient satisfaction and its key drivers. PRW data enable both satisfaction analyses and implications on the level of the individual physician as well as satisfaction analyses and implications on an overall level.

**Objective:**

This study aimed to identify linear and nonlinear effects of patients’ perceived quality of physician appointment service attributes on the overall evaluation measures that are published on PRWs.

**Methods:**

We analyzed large-scale survey data from a German PRW containing 84,680 surveys of patients rating a total of 7038 physicians on 24 service attributes and 4 overall evaluation measures. Elasticities are estimated from regression models with perceived attribute quality as explanatory variables and overall evaluation measures as dependent variables. Depending on the magnitude of the elasticity, service attributes are classified into 3 categories: attributes with diminishing, constant, or increasing returns to overall evaluation.

**Results:**

The proposed approach revealed new insights into what patients value when visiting physicians and what they take for granted. Improvements in the physicians’ pleasantness and friendliness have increasing returns to the publicly available overall evaluation (b=1.26). The practices’ cleanliness (b=1.05) and the communication behavior of a physician during a visit (b level between .97 and 1.03) have constant returns. Indiscretion in the waiting rooms, extended waiting times, and a lack of modernity of the medical equipment (b level between .46 and .59) have the strongest diminishing returns to overall evaluation.

**Conclusions:**

The categorization of the service attributes supports physicians in identifying potential for improvements and prioritizing resource allocation to improve the publicly available overall evaluation ratings on PRWs. Thus, the study contributes to patient-centered health care management and, furthermore, promotes the utility of PRWs through large-scale data analysis.

## Introduction

### Background

Patients are taking a more active role in the decision-making process concerning their medical care [1] in the face of a changing patient-physician relationship [2]. With the World Wide Web as an important source of health-related information [3-5], physician-rating websites (PRWs) are on the rise [6-10], offering an “interesting new source of information about quality of care from the patient's perspective” [11]. PRWs offer the possibility to rate a service encounter with a physician on a Web-based platform. Patients can either post an evaluation to provide feedback to others about their own experience with a physician or read evaluations of other patients before choosing a physician [6].

From a physician’s point of view, PRWs are important because patients’ perceptions of the physician’s service quality are made publicly available. This fact substantially increases the relevance of patient satisfaction to generate positive word of mouth [7,8]. At the same time, patients’ evaluations on PRWs offer directions for the improvement of a physician’s service quality. The ability to identify how and to which extent different service attributes contribute to patients’ overall satisfaction with a physician is of high importance for the physician and health care sector [12].

The emergence of PRWs offers new avenues to analyze patient satisfaction and its key drivers. Existing studies on key drivers of patient satisfaction are usually based on small sample sizes [13,14]. From a key driver perspective, analyzing data from PRWs allows for large-scale analyses based on a large number of patients and physicians to identify how specific service attributes contribute to overall patient satisfaction and patients’ behavioral intentions. Thus, the main purpose of this paper was to conduct a key driver analysis using a multiattribute model applied to large-scale data from a PRW. The specific knowledge about the relationship between a service attribute and the overall evaluation can direct the stakeholders’ efforts to improve performance and set priorities in satisfaction management [15] and help to properly allocate scarce resources [16]. Hence, the findings from our study will strengthen the understanding of patient satisfaction and contribute to the body of knowledge in health care management.

### Usage and Usefulness of Physician-Rating Websites

A broad base of literature has been published so far to investigate PRWs by researchers in many countries worldwide, such as Germany [17-22], Great Britain [23-30], Switzerland [31-33], the Netherlands [34], the United States [6,35-40], Canada [41], and China [42-46]. Rothenfluh and Schulz [31] identified and analyzed 143 PRWs in German- and English-speaking countries (12 countries in total). Apart from the delivery of factual information, such as opening hours and directions [47], PRWs invite patients to evaluate their physicians and articulate their experiences based on the quality of care they received during a medical appointment. Patients produce user-generated content in this vein by posting comments and ratings [48]. From the patients’ point of view, PRWs are a convenient method to share information about the medical care they have received [38,49]. Usage of PRWs is on the rise [9,18,24,49-52], although slowly compared with rating websites in other domains of everyday life [19,33].

Several points of criticism, however, can be addressed toward PRWs. The literature is inconsistent with regard to the link between information revealed on rating sites and quality of care [11,35,40,49,53,54]. Although people may feel challenged to judge a physician’s competence [32,55], they do assess physicians’ competence on PRWs [32].

Text mining approaches [42,43] have been used to analyze what patients articulate in free-text comments. These methods often only identify the most frequently mentioned aspects in the comments while neglecting entirely those that only a minority of reviewers mentioned [43,56]. In addition, as Hao and Zhang [43] underline, people may refrain from posting negative textual comments because of data privacy concerns. Thus, some PRWs deliberately refrain from free-text responses and instead rely on rating scales as answer options (such as the PRWs used in this study).

Holliday et al [50] pinpoint that there are mainly 2 different types of PRWs with regard to the data collection: (1) the so-called *independent websites* (p 626), such as Healthgrades [57], which are run by private companies and nurtured by crowd-sourced data, and (2) PRWs that can be established by health systems, which collect ratings from patients with recent physician or hospital visits (so-called *health system websites*, [50]). The PRW we use in our study can be classified as a special form of *health system website*, as it is run by a noncommercial foundation in cooperation with the main national insurance carriers in Germany. This guarantees that only patients covered by the main national insurance carriers are allowed to post reviews, thus guarding against fraud, which is often seen as another general drawback of PRWs [11].

Information posted on PRWs and especially data in numerical rating scales can be valuable for different stakeholders. Information delivered on PRWs should be of interest not only for patients but also for physicians and other health care providers. Finally, information based on PRW data can help (noncommercial) PRW providers to justify their business model.

### A Key Driver Analysis of Patient Satisfaction Using Physician-Rating Website Data

Customer-oriented health care management and patient satisfaction have become important for physicians in their attempt to attract patients in an increasingly competitive environment [58,59]. Our research about patient satisfaction draws upon the literature on customer satisfaction [60]. There is a broad consensus in research that customer satisfaction with products or services is determined by comparing the previous expectations with the actual perceived performance of the product or service (the so-called expectancy disconfirmation framework [61]). As patients’ expectations often prove to be latent over time and individuals do not consciously compare expectations and actual perceived performance [62], research often follows a performance-only appraisal when studying satisfaction [62,63]. In our study, we followed this argument by assuming that customers (ie, patients) form their overall attitude toward the service experience (ie, appointment with a physician) based on the perceived service quality without a conscious comparison with expectations [64,65]. Furthermore, following the study by Wilkie and Pessemier [66], we used a multiattribute model [67] and assumed that the overall attitude toward a service is the sum of attitudes toward the different attributes of the service. Our approach also allowed us to take into account linear and nonlinear effects of perceived service quality on overall evaluation [68,69], ie, service attributes can have diminishing, constant, or increasing returns.

Diminishing returns mean that improvements in perceived attribute quality have a positive impact on overall evaluation but to a decreasing extent. This means that the contribution to an increase in the overall evaluation gets smaller with increasing perceived attribute quality (following a monotonically increasing and concave function). These service attributes are labeled *basic factors* in the denotation of the 3-factor structure of customer satisfaction [70,71] and are typically taken for granted by patients.

Constant returns mean that improvements in perceived attribute quality have a positive impact on overall evaluation and that the contribution to the improvements remains the same along the scale of possible levels of perceived attribute quality (following a monotonically increasing and linear function). Service attributes with constant returns are denominated as *performance factors* [70,71].

Increasing returns hold that improvements in perceived attribute quality have a positive impact on overall evaluation, but now the contribution to an increase in the overall evaluation expands in size with increasing perceived attribute quality (following a monotonically increasing and convex function). Service attributes with increasing returns are denominated as *excitement factors* [70,71].

Beyond satisfaction or any kind of overall attitude, perceived service quality can also influence repeated purchases [72] and induce positive word of mouth [73]. Consequently, we expanded our multiattribute model of patient satisfaction by using 4 different measures of overall evaluation: (1) overall impression of the physician, (2) patients’ experience with the results of medical treatment by the physician, (3) willingness to recommend the physician, and (4) willingness to revisit the physician for medical treatment.

To the best of our knowledge, no study has conducted a key driver analysis of patient satisfaction using online physician-rating data and thus has taken a comprehensive perspective on the utility of PRWs. Our study aimed to fill this research gap.

## Methods

### Data Sources and Measures

In our study, we used the database of the German noncommercial PRW *Weisse Liste* [74]. This German PRW can be seen as a best practice example with regard to its compliance with quality criteria required for *good* physician-rating portals according to the German Agency for Quality in Medicine [75]. The purpose of this platform is the online provision of physician ratings in terms of perceived attribute quality by actual patients. To initiate the formation of a large base of ratings, the platform sent out the physician-rating survey through its statutory health insurance partners by mail in several waves until autumn 2013. The target group of the mail survey was a representative sample of patients from 2 of the largest statutory health insurances in Germany, and patients were allowed to fill out the physician-rating survey either online or offline. The offline ratings were then transferred to the Web-based PRW. Hence, data from this period contain physician evaluations that are based on either online ratings or ratings via a postal mail survey (offline). The idea behind surveying online and offline at the same time was to gain additional momentum for the data collection process to quickly reach a broad rating database. This approach thus led to a highly representative sample of patients’ ratings (ie, both online and offline segments were able to participate). To participate, patients had to state their name, health insurance carrier, and insurance number. For data protection reasons, physician ratings and patient data were processed separately for both types of data collection. Patients could rate the same physician several times, but only the most recent rating was used in the evaluation.

The online and offline surveys were identical. In the survey, patients were asked several questions related to the following service dimensions: office and staff, communication, and medical treatment by the chosen physician. The questions were worded in the form of 24 different statements, which can be answered on a 4-point scale with 1 as *strongly disagree* to 4 as *strongly agree* and the option to answer *cannot be assessed* (the 24 statements are listed in Table 1 under service attributes). In addition to the service attributes, the following 4 measures of overall evaluation were collected in the survey: “What is your overall impression of this physician?” (*overall impression*), “How would you describe your experience with the results of medical treatment by this physician?” (*experience with results*), “Would you recommend this physician to your best friend?” (*willingness to recommend*), and “Would you visit this physician again, if you had to be medically treated?” (*willingness to revisit*). The 4 measures of overall evaluation were surveyed using a 5-point scale with 1 as *bad* to 5 as *excellent* for *overall impression* and *experience with results* and with 1 as *definitely not* to 5 as *definitely* for *willingness to recommend* and *willingness to revisit*. We rescaled the overall evaluations to a 4-point scale for similarity to the measures of perceived attribute quality. Higher values in the ratings are associated with higher satisfaction for the respective service attribute and a more positive assessment of the overall evaluation measure. Summary scores of the overall evaluations were published on the PRW as soon as 5 or more completed surveys were registered for a physician. We used the same criteria of a minimum of 5 completed surveys for inclusion of physicians in our analysis to avoid biased evaluations by small numbers of surveys.

**Table 1:** Means and standard deviations of the service attributes and overall evaluations.

Service attributes and overall evaluations		Values, mean (SD)
**Service attributes**		
	The physician has a pleasant and friendly manner	3.84 (0.45)
	The physician listens to me carefully	3.77 (0.55)
	The physician handles my questions, concerns, and fears in an empathetic way	3.74 (0.58)
	The physician’s office is clean and neat	3.85 (0.39)
	The physician indicates clearly how to take prescribed medication	3.80 (0.48)
	The physician explains diagnoses, causes, and treatments so that I understand everything	3.72 (0.58)
	The physician does not hurry during the medical treatment	3.68 (0.61)
	I have the impression that the physician will refer me to a specialist if this is medically necessary	3.78 (0.52)
	The physician explains exactly the benefits and associated risks of proposed medical treatments	3.67 (0.62)
	Personal medical records are handled with confidentiality	3.78 (0.48)
	In case of disease, the physician explains various treatment options	3.64 (0.65)
	The physician involves me in decisions about upcoming examinations and treatments	3.66 (0.63)
	The physician conducts physical examinations of me thoroughly	3.63 (0.66)
	The physician’s office creates a well-organized impression	3.70 (0.57)
	The protection of my privacy is respected in the office	3.74 (0.53)
	The staff makes me feel welcome	3.71 (0.56)
	The physician’s office is nicely decorated	3.63 (0.58)
	Consultation time and absences are clearly communicated	3.75 (0.52)
	The physician regularly enquires about my tolerance of the prescribed medication	3.40 (0.81)
	The period between the first contact and the medical appointment is appropriate	3.57 (0.65)
	The medical equipment in the office creates a modern impression	3.37 (0.71)
	The waiting time before entering the physician’s office is adequate	3.38 (0.76)
	The waiting area offers enough space to maintain a distance from other patients	3.33 (0.77)
	Mentioning the reason for my visit in front of other patients is avoided	3.50 (0.74)
**Overall evaluations**		
	Overall impression	3.11 (0.85)
	Experience with results	2.98 (0.86)
	Willingness to recommend	3.57 (0.82)
	Willingness to revisit	3.80 (0.62)

In summary, we had access to a representative random sample containing 84,680 surveys of patients rating a total of 7038 general practitioners collected up to September 2014 (the PRW was launched in May 2011). The number of completed surveys for each physician is between 5 as a minimum and 82 as maximum. In the sample, the average number of completed surveys for each physician is 12 (SD 7). In Table 1, we summarize the means and standard deviations of the measures described previously. We treated the answer *cannot be assessed* as missing values for the service attributes in all subsequent analyses.

### Statistical Analysis

A number of methods for identification of the 3-factor structure of customer satisfaction have been developed and applied outside health care research (for a review of these methods and their application, see the study by Arbore and Busacca [70]). The most widespread approach is the Penalty-Reward-Contrast Analysis introduced by Brandt [76]. One major criticism of this approach is the necessity to dichotomize the rating scales for the perceived attribute quality. Hereby, dummy variables are used only for low and high values of the measures to assess the nonlinear relationship between the perceived attribute quality and the overall evaluation (basic, performance, and excitement factors). This approach has been criticized because of the loss of information caused by dichotomizing the ends of the scale [77,78], but furthermore has to be linked to underestimation of effect sizes and an increased probability of type 2 errors [79].

We used log-log regression models for our analyses. This modeling approach draws from econometric models of demand [80]. The slope coefficient from a log-log regression model identifies if an explanatory variable (ie, perceived attribute quality) has diminishing, constant, or increasing returns to a dependent variable (ie, overall evaluation). These 3 types of relationships have the previously described similarity to the 3-factor structure of customer satisfaction (see the study by Matzler and Sauerwein [71] for basic, performance, and excitement factors).

To empirically identify the 3 different types of response patterns (ie, diminishing, constant, or increasing returns) using the log-log regression model, we first took the natural logarithm (*ln*) on both sides of a linear equation [80]: *ln Y=b_0_+b_1_ ln X*. Then, we estimated the parameters using ordinary least squares and *b_1_* becomes the elasticity of *Y* with regard to *X* (ie, the percentage change in *Y* caused by a one percentage change in *X*, see the study by Varian [81]). The log-log regression model arrives at constant elasticities. This means that the magnitude of the elasticity obtained from our model is independent of the magnitudes of *Y* and *X*.

Depending on the magnitude of the parameter estimate *b_1_,* we can empirically determine the type of (nonlinear) relationship between *X* and *Y*. If *b_1_*<1, the functional relationship is concave, and the attribute measured in *X* has diminishing returns to *Y* (ie, overall evaluation). If *b_1_*=1, the functional relationship is linear, with *X* having constant returns, and if *b_1_*>1, then the functional relationship is convex, where *X* has increasing returns. Applying the log-log regression model with perceived attribute quality as *X* and the measures of overall evaluation as *Y* (both transformed using the natural logarithm) allowed us to classify the service attributes into these 3 categories depending on the magnitude of *b_1_*. We used significance testing with H_0_: *b_1_*=1 to support the classification beyond the sole interpretation of the magnitude of *b_1_*. The parameter *b_0_* serves as an intercept to account for the baseline level of *ln* *Y* (ie, the overall evaluation). We estimated log-log regression models with each service attribute in a single equation to allow for a different starting point of the curve for each *X*. This enables the functional relationships to be more flexibly positioned within the relationship between the perceived attribute quality and the overall evaluation. As each physician in the underlying database has at least 5 ratings for the service attributes and overall evaluations, we used physician-specific intercepts to further account for unobserved heterogeneity (so-called fixed effects, see Baltagi [82]). Therefore, the different intercepts in our model allowed for a different starting point of each service attribute as well as for each physician.

Importantly, our proposed approach of a multiattribute model with nonlinear slope coefficients held a number of relevant assumptions. First, following previous research [83,84], we emphasized that the numerical ratings for our evaluation measures have to be assumed at ratio scale level. From this, our approach asked for a specific coding of the ratings to numerical data. The ratings have to be coded with 1 as the lowest possible numerical value and larger values that increase by 1 unit for each larger rating option. This setting is necessary for the data transformation using the natural logarithm in combination with our assumption of ratio scale level for the ratings to arrive at meaningful nonlinear slope coefficients. Any other coding will make the log-log regression model assume that the numerical values of the ratings are not ratio scale level and that there are values below the minimum when fitting the linear or nonlinear slope (eg, when coding 11-14 instead of 1-4, the estimates would take the range from 1 to 14 into account). Consequently, it is important to mention that our elasticities have to be interpreted within the range of *X* and *Y* used in our data. As usual in multiattribute models, we also assumed that all slope coefficients are positive and monotonically increasing.

To test the robustness of the results from our approach, additional calculations were carried out: to show that our empirical findings do not rely on the log-log regression model only, we analyzed the data with 2 alternative approaches (the results of the robustness checks are available on request). In our first robustness check, we estimated elasticities from a standard linear-linear regression model by multiplying the resulting linear slope coefficients with the ratio of *X* and *Y* (=*b_1_*×[*X*/*Y*]) [80,81]. This approach does not result in constant elasticities but rather elasticities as a function of the values of *X* and *Y* (although we think that the latter is a less meaningful assumption). To show a comparison between these 2 approaches for elasticities in regression analysis, we computed the average elasticities from such a linear-linear regression model. Comparing these results shows that both approaches lead to the same classification (although there is aggregation bias in the linear-linear model because of using the average value across *X* and *Y* ratios). Importantly, the proportion of explained variance (R^2^) is systematically larger for the log-log regression models (our approach) compared with the linear-linear regression models (alternative approach). This outcome supports the position that our log-log regression model should be preferred for 2 reasons: (1) better fit to the data (in general for a log-log regression model with linear and nonlinear slopes compared with a linear-linear regression model with only linear slopes) and (2) more meaningful assumption because of constant elasticities. In our second robustness check, we employed dummy variable coding (with 1 as baseline) for each service attribute as explanatory variable *X.* This leads to 3 dummy variables for the rating values 2, 3, and 4. Using the overall evaluations as dependent variable, the slope coefficients of each of these dummy variables (and each service attribute as *X*) describe the average increase of the dependent variable as a function of the respective rating value compared with the lowest value (=1). Comparing the increase in the 3 slope coefficients for the 3 dummy variables (2 vs 1, 3 vs 1, and 4 vs 1) allowed us to detect diminishing, constant, or increasing returns to scale. If the average of the 2 slope coefficients *2 versus 1* and *4 versus 1* is below, equal, or above the slope coefficient of *3 versus 1*, then this service attribute can be classified as having diminishing, constant, or increasing returns to the overall evaluations. Applying this approach led to the same classification as our approach using the log-log regression model. However, the dummy variable approach showed lower R^2^ compared with the log-log regressions models. In addition, the hypothesis testing cannot be carried out using 1 slope coefficient but has to be combined using 3 slope coefficients. This impedes straightforward hypothesis testing for the 3-factor model of customer satisfaction, which we employed in our research.

## Results

### Parameter Estimates and Model Diagnostics

To assess the relationship between each service attribute and each overall evaluation, we estimated the log-log regression models with each service attribute in a single equation as proposed in the Methods section. This procedure offers 2 further advantages besides providing a different starting point of the curve for each service attribute and each physician. First, because of many service attributes in our setting that are correlated in their ratings, 1 multiple regression equation will produce severe multicollinearity problems. Second, as the answers *cannot be assessed* are flagged as missing values, using several explanatory variables at the same time will lead to case-wise deletion if just one of the service attributes has a missing value in their evaluation. Therefore, estimating models with each service attribute as a single explanatory variable will furthermore allow usage of all available information because of the pairwise consideration of nonmissing values of perceived attribute quality and the overall evaluations. Tables 2-5 provide the summary statistics of our log-log regression models. We provide the parameter estimates of *b_1_* in Tables 2-5 along with the 95% CI for testing the hypothesis H_0_: *b_1_*=1. Bootstrapping is used for hypothesis testing to avoid biased standard errors because of the large sample size [85]. Therefore, we employed 1000 bootstrap replicates and show the 95% CI of this distribution in Tables 2-5 (*bootstrapped [bs] 95% CI*).

Classification of a service attribute depends on 2 determinants: the size of *b_1_* (below 1, around 1, or above 1) and its location with regard to the bs 95% CI. A *b_1_*<1 together with a *bs 95% CI* that does not include 1 classifies the corresponding service attribute as having diminishing returns to the overall evaluation. A *b_1_*>1 together with a *bs 95% CI* that does not include 1 classifies the service attribute as having increasing returns. Estimates of *b_1_* around 1 with a *bs 95% CI* that includes 1 lead to a classification of the corresponding service attribute as having constant returns to the overall evaluations. Table 2 presents the *b_1_* values for all of the 24 service attributes and the overall evaluation criterion of *overall impression* and Table 3 for the overall evaluation criterion of *experience with results*. Table 4 provides the *b_1_* values for all the 24 service attributes and the overall evaluation criterion of *willingness to recommend* and Table 5 for the overall evaluation criterion of *willingness to revisit*. We list the parameter estimates and service attributes in Tables 2-5 in descending order of *b_1_* for the models with *overall impression* as dependent variable. In all of the four tables (Table 2-5) we also present the proportion of explained variance (*R^2^*) and the number of observations used for estimation (*N*).

**Table 2:** Parameter estimates and model diagnostics of log-log regression models 1 (sorted by the size of b).

Service attributes	Overall impression		
	b (bs^a^ 95% CI)	R^2^	Number of observations used, N
The physician has a pleasant and friendly manner	1.26 (1.19-1.34)	0.33	83,697
The physician listens to me carefully	1.09 (1.05-1.14)	0.4	82,887
The physician handles my questions, concerns, and fears in an empathetic way	1.05 (1.02-1.09)	0.43	83,509
The physician’s office is clean and neat	1.05 (0.97-1.15)	0.14	82,924
The physician indicates clearly how to take prescribed medication	1.03 (0.97-1.09)	0.27	82,697
The physician explains diagnoses, causes, and treatments so that I understand everything	1.01 (0.97-1.05)	0.38	83,341
The physician does not hurry during the medical treatment	.97 (0.93-1.01)	0.39	83,393
I have the impression that the physician will refer me to a specialist if this is medically necessary	.93 (0.87-0.99)	0.27	83,123
The physician explains exactly the benefits and associated risks of proposed medical treatments	.92 (0.88-0.95)	0.39	81,536
Personal medical records are handled with confidentiality	.88 (0.81-0.96)	0.19	82,541
In case of disease, the physician explains various treatment options	.87 (0.84-0.91)	0.4	80,961
The physician involves me in decisions about upcoming examinations and treatments	.87 (0.84-0.91)	0.37	80,343
The physician conducts physical examinations of me thoroughly	.86 (0.82-0.90)	0.38	82,936
The physician’s office creates a well-organized impression	.85 (0.80-0.91)	0.25	82,937
The protection of my privacy is respected in the office	.82 (0.75-0.88)	0.21	82,095
The staff makes me feel welcome	.80 (0.74-0.86)	0.22	83,254
The physician’s office is nicely decorated	.72 (0.66-0.77)	0.16	82,928
Consultation time and absences are clearly communicated	.71 (0.66-0.77)	0.15	81,931
The physician regularly enquires about my tolerance of the prescribed medication	.63 (0.60-0.66)	0.34	79,255
The period between the first contact and the medical appointment is appropriate	.59 (0.53-0.63)	0.17	81,183
The medical equipment in the office creates a modern impression	.59 (0.55-0.63)	0.21	74,618
The waiting time before entering the physician’s office is adequate	.52 (0.48-0.56)	0.18	82,839
The waiting area offers enough space to maintain a distance from other patients	.46 (0.43-0.50)	0.15	82,948
Mentioning the reason for my visit in front of other patients is avoided	.46 (0.42-0.50)	0.14	81,753

^a^bs: bootstrapped.

**Table 3:** Parameter estimates and model diagnostics of log-log regression models 2 (sorted by the size of b for overall impression).

Service attributes	Experience with results		
	b (bs^a^ 95% CI)	R^2^	Number of observations used, N
The physician has a pleasant and friendly manner	1.18 (1.10-1.26)	0.27	83,625
The physician listens to me carefully	1.06 (1.01-1.11)	0.35	82,824
The physician handles my questions, concerns, and fears in an empathetic way	1.02 (0.98-1.07)	0.38	83,450
The physician’s office is clean and neat	1.03 (0.94-1.13)	0.13	82,857
The physician indicates clearly how to take prescribed medication	1.04 (0.98-1.10)	0.26	82,645
The physician explains diagnoses, causes, and treatments so that I understand everything	1.00 (0.95-1.04)	0.36	83,277
The physician does not hurry during the medical treatment	.95 (0.91-0.99)	0.35	83,332
I have the impression that the physician will refer me to a specialist if this is medically necessary	.94 (0.89-1.01)	0.26	83,068
The physician explains exactly the benefits and associated risks of proposed medical treatments	0.92 (0.88-0.96)	0.37	81,490
Personal medical records are handled with confidentiality	.87 (0.80-0.94)	0.18	82,483
In case of disease, the physician explains various treatment options	.88 (0.85-0.92)	0.38	80,922
The physician involves me in decisions about upcoming examinations and treatments	.87 (0.84-0.91)	0.35	80,298
The physician conducts physical examinations of me thoroughly	.87 (0.84-0.91)	0.37	82,877
The physician’s office creates a well-organized impression	.83 (0.78-0.89)	0.23	82,883
The protection of my privacy is respected in the office	.80 (0.74-0.86)	0.19	82,034
The staff makes me feel welcome	.78 (0.72-0.83)	0.2	83,188
The physician’s office is nicely decorated	.71 (0.65-0.76)	0.15	82,860
Consultation time and absences are clearly communicated	.72 (0.66-0.78)	0.15	81,879
The physician regularly enquires about my tolerance of the prescribed medication	.64 (0.61-0.67)	0.34	79,206
The period between the first contact and the medical appointment is appropriate	.60 (0.55-0.64)	0.16	81,133
The medical equipment in the office creates a modern impression	.60 (0.56-0.64)	0.2	74,581
The waiting time before entering the physician’s office is adequate	.52 (0.49-0.56)	0.17	82,779
The waiting area offers enough space to maintain a distance from other patients	.47 (0.43-0.50)	0.14	82,888
Mentioning the reason for my visit in front of other patients is avoided	.46 (0.42-0.50)	0.13	81,703

^a^bs: bootstrapped.

**Table 4:** Parameter estimates and model diagnostics of log-log regression models 3 (sorted by the size of b for overall impression).

Service attributes	Willingness to recommend		
	b (bs^a^ 95% CI)	R^2^	Number of observations used, N
The physician has a pleasant and friendly manner	1.33 (1.25-1.42)	0.37	83,786
The physician listens to me carefully	1.16 (1.11-1.21)	0.46	82,978
The physician handles my questions, concerns, and fears in an empathetic way	1.12 (1.08-1.17)	0.49	83,606
The physician’s office is clean and neat	1.08 (0.99-1.17)	0.15	83,022
The physician indicates clearly how to take prescribed medication	1.09 (1.03-1.15)	0.31	82,795
The physician explains diagnoses, causes, and treatments so that I understand everything	1.06 (1.02-1.11)	0.43	83,445
The physician does not hurry during the medical treatment	1.00 (0.96-1.05)	0.42	83,480
I have the impression that the physician will refer me to a specialist if this is medically necessary	1.00 (0.94-1.05)	0.31	83,212
The physician explains exactly the benefits and associated risks of proposed medical treatments	.96 (0.92-1.01)	0.44	81,633
Personal medical records are handled with confidentiality	.91 (0.83-0.99)	0.21	82,628
In case of disease, the physician explains various treatment options	.92 (0.89-0.96)	0.45	81,056
The physician involves me in decisions about upcoming examinations and treatments	.92 (0.88-0.95)	0.42	80,432
The physician conducts physical examinations of me thoroughly	.90 (0.86-0.94)	0.41	83,025
The physician’s office creates a well-organized impression	.91 (0.85-0.97)	0.29	83,033
The protection of my privacy is respected in the office	.85 (0.79-0.91)	0.23	82,173
The staff makes me feel welcome	.87 (0.81-0.93)	0.26	83,349
The physician’s office is nicely decorated	.74 (0.68-0.79)	0.17	83,020
Consultation time and absences are clearly communicated	.74 (0.68-0.81)	0.17	82,028
The physician regularly enquires about my tolerance of the prescribed medication	.64 (0.61-0.68)	0.37	79,346
The period between the first contact and the medical appointment is appropriate	.60 (0.55-0.65)	0.18	81,268
The medical equipment in the office creates a modern impression	.58 (0.53-0.62)	0.2	74,712
The waiting time before entering the physician’s office is adequate	.54 (0.50-0.59)	0.2	82,932
The waiting area offers enough space to maintain a distance from other patients	.47 (0.43-0.51)	0.16	83,040
Mentioning the reason for my visit in front of other patients is avoided	.47 (0.43-0.51)	0.15	81,854

^a^bs: bootstrapped.

**Table 5:** Parameter estimates and model diagnostics of log-log regression models 4 (sorted by the size of b for overall impression).

Service attributes	Willingness to revisit		
	b (bs^a^ 95% CI)	R^2^	Number of observations used, N
The physician has a pleasant and friendly manner	1.03 (0.96-1.10)	0.37	83,757
The physician listens to me carefully	.87 (0.82-0.92)	0.43	82,938
The physician handles my questions, concerns, and fears in an empathetic way	.84 (0.79-0.88)	0.46	83,572
The physician’s office is clean and neat	.77 (0.68-0.85)	0.13	83,008
The physician indicates clearly how to take prescribed medication	.79 (0.73-0.85)	0.28	82,767
The physician explains diagnoses, causes, and treatments so that I understand everything	.76 (0.72-0.81)	0.38	83,421
The physician does not hurry during the medical treatment	.72 (0.67-0.77)	0.36	83,449
I have the impression that the physician will refer me to a specialist if this is medically necessary	.74 (0.68-0.80)	0.29	83,198
The physician explains exactly the benefits and associated risks of proposed medical treatments	.68 (0.63-0.73)	0.38	81,604
Personal medical records are handled with confidentiality	.67 (0.59-0.74)	0.19	82,622
In case of disease, the physician explains various treatment options	.65 (0.60-0.69)	0.38	81,032
The physician involves me in decisions about upcoming examinations and treatments	.65 (0.61-0.70)	0.37	80,410
The physician conducts physical examinations of me thoroughly	.62 (0.57-0.66)	0.33	83,004
The physician’s office creates a well-organized impression	.65 (0.59-0.71)	0.25	83,016
The protection of my privacy is respected in the office	.61 (0.55-0.67)	0.2	82,170
The staff makes me feel welcome	.64 (0.58-0.70)	0.24	83,325
The physician’s office is nicely decorated	.50 (0.45-0.55)	0.14	83,001
Consultation time and absences are clearly communicated	.52 (0.46-0.58)	0.14	82,007
The physician regularly enquires about my tolerance of the prescribed medication	.41 (0.38-0.45)	0.27	79,322
The period between the first contact and the medical appointment is appropriate	.40 (0.36-0.45)	0.14	81,262
The medical equipment in the office creates a modern impression	.36 (0.32-0.40)	0.14	74,689
The waiting time before entering the physician’s office is adequate	.35 (0.32-0.40)	0.14	82,914
The waiting area offers enough space to maintain a distance from other patients	.30 (0.26-0.34)	0.11	83,035
Mentioning the reason for my visit in front of other patients is avoided	.32 (0.28-0.36)	0.11	81,826

^a^bs: bootstrapped.

### Overall Impression and Experience With Results

On the one hand, for *overall impression,* the first 3 service attributes in Table 2 show increasing returns, ie, *b_1_*>1 together with a *bs 95% CI* that does not include 1. These are, in descending order of *b_1_*, “The physician has a pleasant and friendly manner,” “The physician listens to me carefully,” and “The physician handles my questions, concerns, and fears in an empathetic way.” On the other hand, the service attributes “The physician’s office is clean and neat,” “The physician indicates clearly how to take prescribed medication,” “The physician explains diagnoses, causes, and treatments so that I understand everything,” and “The physician does not hurry during the medical treatment” show constant returns to *overall impression* as a dependent variable, ie, values of *b_1_* around 1 together with a *bs 95% CI* that includes 1. All other service attributes have diminishing returns to *overall impression*, that is, they show values of *b_1_* below 1 together with a bs 95% CI that does not include 1. This pattern is similar for *experience with results* (see Table 3) as overall evaluation except for “The physician handles my questions, concerns, and fears in an empathetic way” with constant instead of increasing returns and “The physician does not hurry during the medical treatment,” which has diminishing instead of constant returns. In addition, the service attribute “I have the impression that the physician will refer me to a specialist if this is medically necessary” has constant returns for the model with *experience with results* as a dependent variable.

### Willingness to Recommend and Willingness to Revisit

For *willingness to recommend*, it can be observed in Table 4 that “The physician has a pleasant and friendly manner,” “The physician listens to me carefully,” and “The physician handles my questions, concerns, and fears in an empathetic way” have increasing returns but also “The physician indicates clearly how to take prescribed medication” and “The physician explains diagnoses, causes, and treatments so that I understand everything” are now added to this list. Here, once more, “The physician’s office is clean and neat” and “The physician does not hurry during the medical treatment” have constant returns, together with “I have the impression that the physician will refer me to a specialist if this is medically necessary” and “The physician explains exactly the benefits and associated risks of proposed medical treatments” when it comes to the *willingness to recommend*. For *willingness to revisit* (see Table 5*)*, only “The physician has a pleasant and friendly manner” has constant returns, whereas all other service attributes have diminishing returns.

## Discussion

### Summary of Results and Comparison With Prior Work

Collecting information reported by patients is necessary to make health care more customer oriented [86]. Consequently, analyzing online physician-rating data contributes to the body of knowledge in health care management. Our study makes an important contribution to this topic. We have access to a large number of online physician ratings, which allow a nuanced view on patient satisfaction. Our research goes beyond patient satisfaction (ie, *overall impression* and *experience with results*) by also looking at subsequent behavioral intentions that have important implications for physicians (ie, *willingness to recommend* and *willingness to revisit*). The empirical findings of our large-scale study are highly valuable for physicians because they identify service attributes that deserve an investment of resources. Analyzing perceived service quality helps to understand what patients think makes a good physician and what they value in addition to what medical training provides [87].

The first important result of our study is that the more patients perceive the physician’s manner as being pleasant and friendly, the better is their overall impression as well as perceived experience with the results of the medical treatment. This relationship also applies to *willingness to recommend* as dependent variable. We demonstrated that improvements in these service attributes have increasing returns to the overall evaluation. Other service attributes with increasing returns with regard to *overall impression,* and *willingness to recommend* are being empathetic and listening carefully. Although previous studies about patient satisfaction [59,68,72,88-91] have also shown high importance of these factors, we can extend these findings by demonstrating that these service attributes have increasing returns. In addition, for *willingness to recommend*, it can be observed that “The physician indicates clearly how to take prescribed medication” and “The physician explains diagnoses, causes, and treatments so that I understand everything” also have increasing returns. Thus, communication behaviors of physicians that increase knowledge for patients have a large potential for increasing recommendation behavior if the service is fulfilled beyond average levels of satisfaction. In this context, it is important to mention that all starting points (ie, intercepts) of the latter service attributes are below the average level. Therefore, not fulfilling these services does, in fact, lead to dissatisfaction, whereas improving the perceived attribute quality above the average level leads to increasing returns to the overall evaluations. Another noteworthy finding is that the explanatory power of these models is considerably high (R^2^ between 0.27 and 0.49). This emphasizes the ability of these service attributes to influence the different overall evaluations.

Van Oerle et al [92] argue that physicians are increasingly constrained by limited time and scarce budgets. This evokes a higher attractiveness of online health communities for patients to share their positive or negative experiences. Hence, physicians are well advised to make the most out of this limited time frame during the consultation. Physicians should consistently be friendly, pleasant, and empathetic and should listen carefully to their patients, despite time pressure and budget constraints. This corresponds with the findings of Berry and Bendapudi [93]. They asked patients by means of telephone interviews to recall the best and worst experiences that come into their minds with clinic doctors. Virtually, all the respondents referred to the physician’s behavior (*the bedside manner*, p 113) instead of the physician’s expertise or technical abilities. Berry and Bendapudi [93] argue that although technical skills are very important, they are more difficult to evaluate. Therefore, interpersonal skills appear to receive greater attention when it comes to evaluating the physician.

Another finding of our study is that the service attribute “The physician’s office is clean and neat” has constant returns with respect to all overall evaluations (except *willingness to revisit*). Interestingly, in previous studies, this service attribute was found to have no significant impact on patient satisfaction [72,90] or a rather weak impact on overall quality evaluation [94]. The reasons for this change in patients’ preference between the studies from the 1990s and this study may be an increased knowledge and concern about the possibility of infections resulting from a visit to health care facilities where sick people congregate. Paddison et al [95] demonstrate this for hospital-based surroundings in which cleanliness plays a very important role for patients because of their concerns about infections. Thus, patients of physicians may transfer similar concerns to the primary care context and may have a higher awareness of infections resulting from a visit. A physician’s clean and neat office may signal to a patient that other patients’ germs and diseases are not transmitted easily. Hence, it should be emphasized that the results of this study indicate that cleanliness has constant returns with potential for improvements to the overall evaluations with the entire satisfaction range of perceived attribute quality.

The results of our study also show that improving communication behaviors of physicians that increase knowledge for patients has constant returns. The service attribute “The physician indicates clearly how to take prescribed medication” and “The physician explains diagnoses, causes, and treatments so that I understand everything” shows constant returns for *overall impression* and *experience with results*. Such an influence on patient satisfaction is in line with previous research [96], as competence in communication is seen as a facet of medical competence [97]. When it comes to *willingness to recommend*, the service attributes “I have the impression that the physician will refer me to a specialist if this is medically necessary” and “The physician explains exactly the benefits and associated risks of proposed medical treatments” have constant returns. Again, the results corroborate previous research, and we are able to emphasize the importance of these service attributes to improve patient satisfaction and their willingness to recommend. Importantly, these models describing the service attributes with constant returns to scale also show considerably high explanatory power (R^2^ between 0.13 and 0.44). Lanjananda and Patterson [98] found significant predictors of nurses’ customer-oriented behavior: basic personality, customer orientation as surface trait, and nurses’ perceptions of the service climate and their commitment to the hospital. Thus, we can conclude that the physician’s personality, patient orientation, and their commitment are also important in explaining the degree of patient-oriented health care service.

The results displayed at the lower end of Tables 2-5 reveal that extended waiting times for medical appointments, a lack of modernity of the medical equipment, and the facilities of the waiting area have diminishing returns. If fulfilled poorly, they are likely to have a strong negative impact on the overall evaluations. The most important of these service attributes with potential for decreased overall evaluation is related to indiscretion in the reception area or the waiting room. If a patient cannot state the reason for the visit without being overheard by others, this is likely to substantially reduce the overall evaluation. Privacy reflects perceptions that a patient’s intimacy may be compromised by the mere presence of others [99]. Respect of privacy was identified as the most important contributor to overall satisfaction by Carlucci et al [68]. This ties in with the results of our study. However, our approach allows for a more nuanced interpretation of the high importance of privacy: given that privacy shows diminishing returns in our results, it is highly likely that patients see privacy as a very basic factor and that lack of privacy leads to strong dissatisfaction. At the same time, the existence of privacy can only lead to average satisfaction but not to high levels of satisfaction with potential for excitement. Our results indicate that the abovementioned service attributes, in particular, privacy in the reception area or the waiting room, are all factors that patients appear to take for granted. Hence, absence or poor-quality levels are likely to substantially reduce overall evaluation, whereas high levels of fulfillment do not further contribute to patients’ overall evaluation.

### Implications for Health Care Management

The number of PRWs is on the rise [8], and PRWs are becoming increasingly popular among patients [6]. Therefore, it is important to provide knowledge about what drives these publicly available overall evaluations. When PRWs collect and present information about patients’ experiences and satisfaction with individual physicians, our proposed approach can help physicians to classify the service attributes with regard to their returns, identify deficits, improve the quality of chosen service attributes, and stimulate improved ratings in the future. Monitoring these service attribute classifications (also over time) is, therefore, an important issue.

Implications from our results for the service attributes with diminishing returns to the overall evaluation are as follows: a physician and his or her staff are well advised to work toward efficient patient scheduling, modern medical equipment, and a generously appointed waiting room to deliver personal space between the patients; and to ensure sufficient discretion at the reception desk to allow patients to state their reason for the visit without being overheard.

If physicians want to improve their measures of overall evaluation on PRWs and aim to stand out from competitors, they are well advised to improve those service attributes that were shown to have constant and increasing returns. Many service attributes have diminishing returns with respect to patients’ overall evaluation of the physician. These factors still have great relevance for patients’ satisfaction because they lead to dissatisfaction if the perceived attribute quality is below the average level. All these service attributes should be seen as expected by patients to be at a satisfactory level, and therefore, delivering these standards is a prerequisite for patient satisfaction. However, further improvement of the perceived attribute quality beyond the average satisfaction level does not lead to substantial increases in overall evaluation because of the diminishing returns.

In line with the claim to protect the *voice of the patient needs* [100], the advantage of using PRWs as a source of patients’ experiences is the condition of anonymity. Everyone posting a review on a PRW—at least in the case of the database we used for our study—can be secure in the knowledge that data privacy is taken seriously and that their evaluation will not influence future contact with the physician, at least on an individual level. On the other hand, the results of this study can be used by physicians to create patient delight—similar to customer delight [101,102], by focusing on the service attributes with increasing returns.

### Limitations

This study has the following limitations that set the stage for future research opportunities. First, it should be recognized that the implications of our study are limited to a fixed set of attributes. This may have the potential to divert physicians’ attention away from other important aspects of health care [59]. Therefore, it is important for future research not only to avoid the exclusion of relevant service attributes but also to account for other aspects of service quality that may not be perceived by patients when it comes to improving health care management. Second, the average values of the perceived service quality are high and show a tendency toward high satisfaction in our sample (see Table 1). Such a response bias is well known in patient satisfaction surveys from Web-based PRWs [9,30,38] and thus is in accordance with these existing studies.

With regard to the time frame of the data collection, the focus was set on the introductory phase of the PRW (May 2011 to September 2014), and the large-scale data were drawn from the PRW in September 2014. In the meantime, patients can only post their rating on the PRW and not offline as was possible during the earlier phase of the PRW. Thus, data available in the introductory phase should cover the evaluation and spectrum of opinions of a large range of patients throughout the whole population. This specific point of data collection, therefore, reflects the broad range of experiences of the patient-physician encounter from a representative sample of the total population quite well (both online and offline population segments). This provides the opportunity to use this initial phase of a PRW’s large-scale data as a reference point for further studies. Especially, a longitudinal setting would deliver fruitful insights into developments of categorization over time, bearing in mind that the introductory phase was also characterized by an additional opportunity for patients to rate physicians through mail. Thus, using the large-scale data, the results of this study deliver an important reference point to monitor patients’ evaluation of physicians over time.

Regardless of the limitations discussed previously, the relevance of all derived implications is still high for health care management because of the fact that all the ratings on PRWs are publicly available and can influence patients in their choice of a physician.

## Abbreviations

bs: bootstrapped
PRW: physician-rating website

## References

[1] Zeithaml VA, Berry LL, Parasuraman A (1993). The nature and determinants of customer expectations of service. J Acad Mark Sci.

[2] Roettl J, Bidmon S, Terlutter R (2016). What predicts patients' willingness to undergo online treatment and pay for online treatment? Results from a web-based survey to investigate the changing patient-physician relationship. J Med Internet Res.

[3] Fox S (2014). Pew Research Center.

[4] Laugesen J, Hassanein K, Yuan Y (2015). The impact of internet health information on patient compliance: a research model and an empirical study. J Med Internet Res.

[5] Bidmon S, Terlutter R (2015). Gender differences in searching for health information on the internet and the virtual patient-physician relationship in Germany: exploratory results on how men and women differ and why. J Med Internet Res.

[6] Hanauer DA, Zheng K, Singer DC, Gebremariam A, Davis MM (2014). Public awareness, perception, and use of online physician rating sites. J Am Med Assoc.

[7] Emmert M, Meier F (2013). An analysis of online evaluations on a physician rating website: evidence from a German public reporting instrument. J Med Internet Res.

[8] Emmert M, Meier F, Pisch F, Sander U (2013). Physician choice making and characteristics associated with using physician-rating websites: cross-sectional study. J Med Internet Res.

[9] Terlutter R, Bidmon S, Röttl J (2014). Who uses physician-rating websites? Differences in sociodemographic variables, psychographic variables, and health status of users and nonusers of physician-rating websites. J Med Internet Res.

[10] Bidmon S, Terlutter R, Röttl J (2014). What explains usage of mobile physician-rating apps? Results from a web-based questionnaire. J Med Internet Res.

[11] Verhoef LM, van de Belt TH, Engelen LJ, Schoonhoven L, Kool RB (2014). Social media and rating sites as tools to understanding quality of care: a scoping review. J Med Internet Res.

[12] Gill L, White L (2009). A critical review of patient satisfaction. Leadersh Health Serv.

[13] Chang WJ, Chang YH (2013). Patient satisfaction analysis: identifying key drivers and enhancing service quality of dental care. J Dent Sci.

[14] Davis J, Burrows JF, Khallouq BB, Rosen P (2017). Predictors of patient satisfaction in pediatric oncology. J Pediatr Oncol Nurs.

[15] Hattenberger G, Pechlaner H, Matzler K (2004). Lifestyle-Typologies and Market Segmentation: The Case of Alpine Skiing Tourism.

[16] Mikulic J, Prebežac D (2011). A critical review of techniques for classifying quality attributes in the Kano model. Manag Serv Qual Int J.

[17] Emmert M, Sander U, Pisch F (2013). Eight questions about physician-rating websites: a systematic review. J Med Internet Res.

[18] McLennan S, Strech D, Meyer A, Kahrass H (2017). Public awareness and use of German physician ratings websites: cross-sectional survey of four north German cities. J Med Internet Res.

[19] McLennan S, Strech D, Kahrass H (2018). Why are so few patients rating their physicians on German physician rating websites? A qualitative study. BMC Health Serv Res.

[20] Emmert M, Sander U, Esslinger AS, Maryschok M, Schöffski O (2012). Public reporting in Germany: the content of physician rating websites. Methods Inf Med.

[21] Emmert M, Sauter L, Jablonski L, Sander U, Taheri-Zadeh F (2017). Do physicians respond to web-based patient ratings? An analysis of physicians' responses to more than one million web-based ratings over a six-year period. J Med Internet Res.

[22] Emmert M, Meszmer N, Sander U (2016). Do health care providers use online patient ratings to improve the quality of care? Results from an online-based cross-sectional study. J Med Internet Res.

[23] Greaves F, Pape UJ, King D, Darzi A, Majeed A, Wachter RM, Millett C (2012). Associations between Web-based patient ratings and objective measures of hospital quality. Arch Intern Med.

[24] Greaves F, Millett C (2012). Consistently increasing numbers of online ratings of healthcare in England. J Med Internet Res.

[25] Greaves F, Ramirez-Cano D, Millett C, Darzi A, Donaldson L (2013). Use of sentiment analysis for capturing patient experience from free-text comments posted online. J Med Internet Res.

[26] Powell J, Boylan AM, Greaves F (2015). Harnessing patient feedback data: a challenge for policy and service improvement. Digit Health.

[27] Greaves F, Ramirez-Cano D, Millett C, Darzi A, Donaldson L (2013). Harnessing the cloud of patient experience: using social media to detect poor quality healthcare. BMJ Qual Saf.

[28] Patel S, Cain R, Neailey K, Hooberman L (2016). Exploring patients' views toward giving web-based feedback and ratings to general practitioners in England: a qualitative descriptive study. J Med Internet Res.

[29] Patel S, Cain R, Neailey K, Hooberman L (2015). General practitioners' concerns about online patient feedback: findings from a descriptive exploratory qualitative study in England. J Med Internet Res.

[30] Galizzi MM, Miraldo M, Stavropoulou C, Desai M, Jayatunga W, Joshi M, Parikh S (2012). Who is more likely to use doctor-rating websites, and why? A cross-sectional study in London. BMJ Open.

[31] Rothenfluh F, Schulz PJ (2018). Content, quality, and assessment tools of physician-rating websites in 12 countries: quantitative analysis. J Med Internet Res.

[32] Rothenfluh F, Schulz PJ (2017). Physician rating websites: what aspects are important to identify a good doctor, and are patients capable of assessing them? A mixed-methods approach including physicians' and health care consumers' perspectives. J Med Internet Res.

[33] Rothenfluh F, Germeni E, Schulz PJ (2016). Consumer decision-making based on review websites: are there differences between choosing a hotel and choosing a physician?. J Med Internet Res.

[34] Damman OC, van den Hengel YK, van Loon AJ, Rademakers J (2010). An international comparison of web-based reporting about health care quality: content analysis. J Med Internet Res.

[35] Emmert M, Adelhardt T, Sander U, Wambach V, Lindenthal J (2015). A cross-sectional study assessing the association between online ratings and structural and quality of care measures: results from two German physician rating websites. BMC Health Serv Res.

[36] Burkle CM, Keegan MT (2015). Popularity of internet physician rating sites and their apparent influence on patients' choices of physicians. BMC Health Serv Res.

[37] Gao GG, McCullough JS, Agarwal R, Jha AK (2012). A changing landscape of physician quality reporting: analysis of patients' online ratings of their physicians over a 5-year period. J Med Internet Res.

[38] Lagu T, Hannon NS, Rothberg MB, Lindenauer PK (2010). Patients' evaluations of health care providers in the era of social networking: an analysis of physician-rating websites. J Gen Intern Med.

[39] Trehan SK, Nguyen JT, Marx R, Cross MB, Pan TJ, Daluiski A, Lyman S (2018). Online patient ratings are not correlated with total knee replacement surgeon-specific outcomes. HSS J.

[40] Chen J, Presson A, Zhang C, Ray D, Finlayson S, Glasgow R (2018). Online physician review websites poorly correlate to a validated metric of patient satisfaction. J Surg Res.

[41] Liu JJ, Matelski JJ, Bell CM (2018). Scope, breadth, and differences in online physician ratings related to geography, specialty, and year: observational retrospective study. J Med Internet Res.

[42] Li J, Liu M, Li X, Liu X, Liu J (2018). Developing embedded taxonomy and mining patients' interests from web-based physician reviews: mixed-methods approach. J Med Internet Res.

[43] Hao H, Zhang K (2016). The voice of Chinese health consumers: a text mining approach to web-based physician reviews. J Med Internet Res.

[44] Hao H, Zhang K, Wang W, Gao G (2017). A tale of two countries: international comparison of online doctor reviews between China and the United States. Int J Med Inform.

[45] Hao H (2015). The development of online doctor reviews in China: an analysis of the largest online doctor review website in China. J Med Internet Res.

[46] Zhang W, Deng Z, Hong Z, Evans R, Ma J, Zhang H (2018). Unhappy patients are not alike: content analysis of the negative comments from China's good doctor website. J Med Internet Res.

[47] Fischer S, Emmert M, Gurtner S, Soyez K (2015). A review of scientific evidence for public perspectives on online rating websites of healthcare providers. Challenges And Opportunities in Health Care Management.

[48] Greaves F, Pape UJ, King D, Darzi A, Majeed A, Wachter RM, Millett C (2012). Associations between internet-based patient ratings and conventional surveys of patient experience in the English NHS: an observational study. BMJ Qual Saf.

[49] Greaves F, Pape UJ, Lee H, Smith DM, Darzi A, Majeed A, Millett C (2012). Patients' ratings of family physician practices on the internet: usage and associations with conventional measures of quality in the English National Health Service. J Med Internet Res.

[50] Holliday AM, Kachalia A, Meyer GS, Sequist TD (2017). Physician and patient views on public physician rating websites: a cross-sectional study. J Gen Intern Med.

[51] McLennan S, Strech D, Reimann S (2017). Developments in the frequency of ratings and evaluation tendencies: A review of German physician rating websites. J Med Internet Res.

[52] Patel S, Cain R, Neailey K, Hooberman L (2018). Public awareness, usage, and predictors for the use of doctor rating websites: cross-sectional study in England. J Med Internet Res.

[53] Totten AM, Wagner J, Tiwari A, O'Haire C, Griffin J, Walker M (2012). Closing the quality gap: revisiting the state of the science (vol. 5: public reporting as a quality improvement strategy). Evid Rep Technol Assess (Full Rep).

[54] Meszmer N, Jaegers L, Schöffski O, Emmert M (2018). Spiegeln Onlinebewertungen Strukturunterschiede in der Versorgung wider? Eine Analyse am Beispiel von Arztbewertungsportalen [Do online ratings reflect structural differences in healthcare? The example of German physician-rating websites]. Z Evid Fortbild Qual Gesundhwes.

[55] Emmert M, Meier F, Heider AK, Dürr C, Sander U (2014). What do patients say about their physicians? An analysis of 3000 narrative comments posted on a German physician rating website. Health Policy.

[56] Guo Y, Barnes SJ, Jia Q (2017). Mining meaning from online ratings and reviews: tourist satisfaction analysis using latent dirichlet allocation. Tour Manag.

[57] Healthgrades.

[58] Otani K, Waterman B, Faulkner KM, Boslaugh S, Burroughs TE, Dunagan WC (2009). Patient satisfaction: focusing on 'excellent'. J Healthc Manag.

[59] Schlesinger M, Grob R, Shaller D (2015). Using patient-reported information to improve clinical practice. Health Serv Res.

[60] Oliver RL (1997). Satisfaction: A Behavioral Perspective On The Consumer.

[61] Oliver RL (1980). A cognitive model of the antecedents and consequences of satisfaction decisions. J Mark Res.

[62] Leisen B, Hyman MR (2004). Antecedents and consequences of trust in a service provider: the case of primary care physicians. J Bus Res.

[63] Mummalaneni V, Gopalakrishna P (1997). Access, resource, and cost impacts on consumer satisfaction with health care: a comparison across alternative health care modes and time. J Bus Res.

[64] Churchill GA, Surprenant C (1982). An investigation into the determinants of customer satisfaction. J Mark Res.

[65] Anderson EW, Sullivan MW (1993). The antecedents and consequences of customer satisfaction for firms. Mark Sci.

[66] Wilkie WL, Pessemier EA (1973). Issues in marketing's use of multi-attribute attitude models. J Mark Res.

[67] van Ittersum K, Pennings JM, Wansink B, van Trijp HC (2007). The validity of attribute-importance measurement: a review. J Bus Res.

[68] Carlucci D, Renna P, Schiuma G (2013). Evaluating service quality dimensions as antecedents to outpatient satisfaction using back propagation neural network. Health Care Manag Sci.

[69] Huang R, Sarigöllü E (2008). Assessing satisfaction with core and secondary attributes. J Bus Res.

[70] Arbore A, Busacca B (2009). Customer satisfaction and dissatisfaction in retail banking: exploring the asymmetric impact of attribute performances. J Retail Consum Serv.

[71] Matzler K, Sauerwein E (2002). The factor structure of customer satisfaction: an empirical test of the importance grid and the penalty-reward-contrast analysis. Int J Serv Ind Manag.

[72] Mittal V, Ross WT, Baldasare PM (1998). The asymmetric impact of negative and positive attribute-level performance on overall satisfaction and repurchase intentions. J Mark.

[73] Harrison-Walker LJ (2001). The measurement of word-of-mouth communication and an investigation of service quality and customer commitment as potential antecedents. J Serv Res.

[74] Weisse Liste.

[75] Fischer S, Gurtner S, Soyez K (2015). Project 'Weisse Liste': a German best practice example for online provider ratings in health care. Challenges and Opportunities in Health Care Management.

[76] Brandt RD (1988). How service marketers can identify value-enhancing service elements. J Serv Mark.

[77] Albayrak T, Caber M (2013). Penalty–Reward-Contrast Analysis: a review of its application in customer satisfaction research. Total Qual Manag Bus Excel.

[78] Sampson SE, Showalter MJ (1999). The performance-importance response function: observations and implications. Serv Ind J.

[79] Rucker DD, McShane BB, Preacher KJ (2015). A researcher's guide to regression, discretization, and median splits of continuous variables. J Consum Psychol.

[80] Hanssens DM, Parsons LJ, Schultz RL (2001). Market Response Models: Econometric and Time Series Analysis.

[81] Varian HR (2010). Intermediate Microeconomics: A Modern Approach. Eighth Edition.

[82] Baltagi B (2013). Econometrics. Fifth Edition.

[83] Michell J (1986). Measurement scales and statistics: a clash of paradigms. Psychol Bull.

[84] Zulueta J, Piscitello A, Rasic M, Easter R, Babu P, Langenecker SA, McInnis M, Ajilore O, Nelson PC, Ryan K, Leow A (2018). Predicting mood disturbance severity with mobile phone keystroke metadata: a BiAffect digital phenotyping study. J Med Internet Res.

[85] Efron B, Tibshirani R (1986). Bootstrap methods for standard errors, confidence intervals, and other measures of statistical accuracy. Stat Sci.

[86] Sofaer S, Firminger K (2005). Patient perceptions of the quality of health services. Annu Rev Public Health.

[87] Jain S (2010). Googling ourselves--what physicians can learn from online rating sites. N Engl J Med.

[88] Bacon N (2009). Will doctor rating sites improve standards of care? Yes. BMJ.

[89] Bartlett EE, Grayson M, Barker R, Levine DM, Golden A, Libber S (1984). The effects of physician communications skills on patient satisfaction; recall, and adherence. J Chronic Dis.

[90] Mittal V, Baldasare PM (1996). Eliminate the negative. Managers should optimize rather than maximize performance to enhance patient satisfaction. J Health Care Mark.

[91] Robbins JA, Bertakis KD, Helms LJ, Azari R, Callahan EJ, Creten DA (1993). The influence of physician practice behaviors on patient satisfaction. Fam Med.

[92] van Oerle S, Mahr D, Lievens A (2016). Coordinating online health communities for cognitive and affective value creation. J Serv Manag.

[93] Berry LL, Bendapudi N (2007). Health care: a fertile field for service research. J Serv Res.

[94] Seibert JH, Strohmeyer JM, Carey RG (1996). Evaluating the physician office visit: in pursuit of a valid and reliable measure of quality improvement efforts. J Ambul Care Manage.

[95] Paddison CA, Abel GA, Roland MO, Elliott MN, Lyratzopoulos G, Campbell JL (2015). Drivers of overall satisfaction with primary care: evidence from the English General Practice Patient Survey. Health Expect.

[96] Comstock L M, Hooper E M, Goodwin J M, Goodwin J S (1982). Physician behaviors that correlate with patient satisfaction. J Med Educ.

[97] Buller MK, Buller DB (1987). Physicians' communication style and patient satisfaction. J Health Soc Behav.

[98] Lanjananda P, Patterson PG (2009). Determinants of customer‐oriented behavior in a health care context. J Serv Manag.

[99] Ponsignon F, Smart A, Williams M, Hall J (2015). Healthcare experience quality: an empirical exploration using content analysis techniques. J Serv Manag.

[100] Elg M, Engström J, Witell L, Poksinska B (2012). Co‐creation and learning in health‐care service development. J Serv Manag.

[101] Rust RT, Oliver RL (2000). Should we delight the customer?. J Acad Mark Sci.

[102] Oliver RL, Rust RT, Varki S (1997). Customer delight: foundations, findings and managerial insight. J Retail.

